# Dispersal and Land Cover Contribute to Pseudorabies Virus Exposure in Invasive Wild Pigs

**DOI:** 10.1007/s10393-020-01508-6

**Published:** 2021-01-14

**Authors:** Felipe A. Hernández, Amanda N. Carr, Michael P. Milleson, Hunter R. Merrill, Michael L. Avery, Brandon M. Parker, Cortney L. Pylant, James D. Austin, Samantha M. Wisely

**Affiliations:** 1 School of Natural Resources and Environment, University of Florida, Gainesville, FL 32611 USA; 2 Present Address: Facultad de Ciencias Veterinarias, Instituto de Medicina Preventiva Veterinaria, Universidad Austral de Chile, Edificio Federico Saelzer, 5º Piso, Campus Isla Teja S/N, Valdivia, Chile; 3 Department of Wildlife Ecology and Conservation, University of Florida, Gainesville, FL 32611 USA; 4 United States Department of Agriculture, Animal and Plant Health Inspection Service, Wildlife Services, Gainesville, FL 32641 USA; 5 Department of Agricultural and Biological Engineering, University of Florida, Gainesville, FL 32611 USA; 6 United States Department of Agriculture, Animal and Plant Health Inspection Service, Wildlife Services, National Wildlife Research Center, Gainesville, FL 32641 USA

**Keywords:** Dispersal, Florida, Landscape composition, Pathogen exposure, PrV, *Sus scrofa*, USA, Wild pigs

## Abstract

We investigated the landscape epidemiology of a globally distributed mammal, the wild pig (*Sus scrofa*), in Florida (U.S.), where it is considered an invasive species and reservoir to pathogens that impact the health of people, domestic animals, and wildlife. Specifically, we tested the hypothesis that two commonly cited factors in disease transmission, connectivity among populations and abundant resources, would increase the likelihood of exposure to both pseudorabies virus (PrV) and *Brucella* spp. (bacterial agent of brucellosis) in wild pigs across the Kissimmee Valley of Florida. Using DNA from 348 wild pigs and sera from 320 individuals at 24 sites, we employed population genetic techniques to infer individual dispersal, and an Akaike information criterion framework to compare candidate logistic regression models that incorporated both dispersal and land cover composition. Our findings suggested that recent dispersal conferred higher odds of exposure to PrV, but not *Brucella* spp., among wild pigs throughout the Kissimmee Valley region. Odds of exposure also increased in association with agriculture and open canopy pine, prairie, and scrub habitats, likely because of highly localized resources within those land cover types. Because the effect of open canopy on PrV exposure reversed when agricultural cover was available, we suggest that small-scale resource distribution may be more important than overall resource abundance. Our results underscore the importance of studying and managing disease dynamics through multiple processes and spatial scales, particularly for non-native pathogens that threaten wildlife conservation, economy, and public health.

**Supplementary Information:**

The online version of this article (10.1007/s10393-020-01508-6) contains supplementary material, which is available to authorized users.

## Introduction

Host ecology is a major contributing factor to the patterns of pathogen emergence across a landscape. The movement of individual animals, particularly dispersal or migration of infected hosts, drives the spread of directly transmitted diseases by introducing a pathogen to naïve populations (Russell et al. 2004; Hosseini et al. 2006; Macdonald and Laurenson 2006; Altizer et al. 2011). As infected hosts disperse across the landscape, they affect the rate of pathogen spread, spatial distribution of infection, and the likelihood of new exposures (Cullingham et al. 2008). Landscape composition influences a host’s exposure to pathogens either by facilitating or hindering contact between individuals or groups (Blanchong et al. 2008; Rees et al. 2008; Root et al. 2009; Barton et al. 2010; Tardy et al. 2018). For example, high rates of dispersal and contact among individual white-tailed deer (*Odocoileus virginianus*) have been suggested as a potential mechanism for long-distance spread of chronic wasting disease (CWD), a prion disease of cervids (Kelly et al. 2010; Cullingham et al. 2011), and of bovine tuberculosis (bTB) among sympatric elk (*Cervus canadensis*) subpopulations (Vander Wal et al. 2013).

Because patterns of host dispersal influence that of pathogens, the identification of environmental variables that influence host movement also contribute to spatial distribution and occurrence of disease agents, vectors, and reservoirs (Ostfeld and LoGiudice 2003; Collinge et al. 2005; Storm et al. 2013; McAlpine et al. 2017), and determine pathogen exposure (Langlois et al. 2001; Riley 2007; Cullingham et al. 2008; Biek and Real 2010; Meentemeyer et al. 2012). Identifying land cover features correlated with the distribution of invasive species carrying non-native pathogens may therefore facilitate protection of both biodiversity and human health via habitat and land use management.

Wild pigs (*Sus scrofa*) are one of the most widely distributed mammals in the world and are considered invasive species on multiple continents, including North America. In the USA, a recent and rapid range expansion has led to the establishment of free-ranging populations in as many as 44 states (Barrios-Garcia and Ballari 2012; Bevins et al. 2014). The rapid spread of wild pigs has been related to both intrinsic (e.g., ability to adapt to diverse habitat types) and extrinsic causes (e.g., human-mediated movement) throughout the whole country (Seward et al. 2004; Bevins et al. 2014). Regionally, a long and continuous history of anthropogenic movement has become the principal source of wild pig introductions and dispersal in places like Florida, which is evident in the high intermixing of wild pigs from different genetic backgrounds (Hernández et al. 2018). However, despite their broad geographic distribution and adaptability, wild pigs have physiological and resource limitations that may influence individual movement patterns and fine-scale distribution (McClure et al. 2015; Snow et al. 2017). Wild pigs depend on habitats with suitable natural or artificial forage resources, and water and cover to thermoregulate during periods of high temperatures (Choquenot and Ruscoe 2003; Mayer and Brisbin Jr 2009). Specifically, hardwood forests provide hard mast (Geisser and Reyer 2005) and shade cover (Choquenot and Ruscoe 2003), and wetland-riparian systems provide important wallowing, cooling, and feeding opportunities (Gaston et al. 2008). Cropland and pastures also concentrate high densities of wild pigs due to their attraction to abundant artificial food resources (Schley and Roper 2003; Herrero et al. 2006). By contrast, open canopy habitats such as Gulf Coast pine forests, wet, and dry prairies, Florida scrubland, and human settlement areas are less frequently utilized by the species, which is likely due to limited cover and resource availability (Mayer and Brisbin Jr 2009; Saito et al. 2012; Keiter and Beasley 2017). Previous studies have explored how the combination of large-scale patterns of dispersal and local patterns of land cover composition affects disease exposure rates on wild pigs (e.g., Cowled et al. 2012; Pearson et al. 2014), contributing to address the growing concern surrounding the spread of pathogens by the species, some of which can severely impact public health, domestic animals, and wildlife (Seward et al. 2004; Meng et al. 2009).

Two infectious pathogens harbored by wild pig populations throughout their global distribution are pseudorabies virus (PrV or Aujeszky’s disease—caused by *Suid alphaherpesvirus*) and *Brucella* spp. (bacterial agent of brucellosis). Both pathogens are directly transmitted by exposure to oro-nasal fluids or sexual contact, and exposure typically leads to lifelong infection accompanied by neutralizing antibodies in wild pigs (Müller et al. 2011; Leiser et al. 2013). Although mortality is rarely associated with either PrV or *Brucella* spp. in adult wild pigs (Müller et al. 2011; Leiser et al. 2013), both pathogens can be lethal to other non-suid species. For example, PrV is lethal to mammalian carnivores and is an emerging health threat to the endangered Florida panther (*Puma concolor coryi*), which preys on wild pigs (Glass et al. 1994). *Brucella* spp. is also a major zoonotic pathogen globally, and *Brucella suis* is one of the most prevalent zoonotic pathogens affecting Floridians (Florida Department of Health 2017). The pathogen produces serious, lifelong health complications in humans if untreated (Franco et al. 2007). Commercial livestock in the USA are considered free of PrV and *Brucella* spp., yet both pathogens are widespread in free-living wild pig populations throughout the country, which increases the risk of reintroduction of the diseases into commercial herds (Pedersen et al. 2012, 2013). Although both PrV and *Brucella* spp. can severely impact wildlife conservation, public health and the livestock industry, little is known about how host-dependent and environmental factors could predict the risk of pathogen exposure in wild pigs. In this regard, further studies are warranted to inform efficient management and control decisions on the spread of wild pigs and diseases at the landscape level.

This study tested two main hypotheses concerning the effect of movement and land cover composition on PrV and *Brucella* spp. exposure among wild pigs across the Kissimmee Valley of Florida (USA). First, because wild pig migration may enhance contact rates between pathogen-exposed and susceptible individuals, we hypothesized that recent dispersal would be predictive of a higher likelihood of PrV and *Brucella* spp. exposure in individual wild pigs. Second, because habitats with high resource availability, like hardwood forests, freshwater wetlands and agriculture, would theoretically support a higher occurrence and density of wild pigs, we hypothesized that animals would exhibit a higher likelihood of pathogen exposure in high resource habitats than in limited resource habitats.

## Methods

### Sample Collection

**Figure 1:**
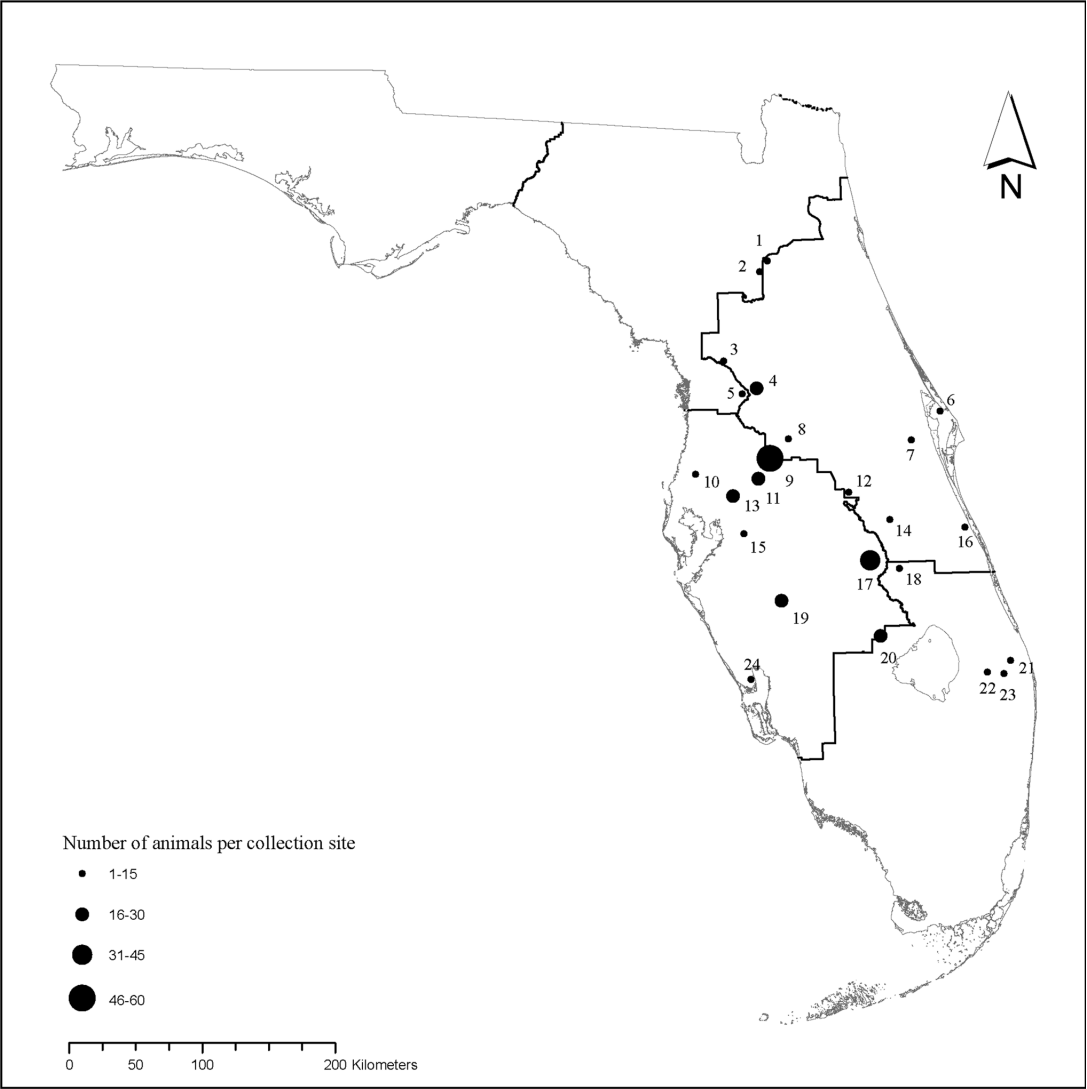
Distribution of wild pig (*Sus scrofa*) collection sites through the Kissimmee Valley of Florida, USA, 2014 to 2016.

Between January 2014 and March 2016, we collected blood and/or hair samples from 348 wild pigs at 24 sites across the Kissimmee Valley of Florida (U.S.) (Fig. 1). We sampled animals opportunistically as part of a national wild pig disease monitoring effort led by the United States Department of Agriculture (USDA), Animal Plant and Health Inspection Service, Wildlife Services, National Wildlife Disease Program. Sampled pigs were either trapped and euthanized during animal control efforts conducted by USDA, or legally harvested by hunters at hunter check stations on federal and state wildlife management areas, military bases, and private properties. We recorded demographic data for each animal, which included sex, age, and sampling location. Specifically, we used body size, reproductive traits, and tooth eruption patterns (Matschke, 1967) to classify animals as adults (≥ 1 year), sub-adults (2 months–1 year), or juveniles (< 2 months). For PrV and *Brucella* spp. serological analyses, we collected up to 35 ml blood from 320 of the 348 wild pigs using 9 ml Covidien^®^ serum separator tubes (Covidien AG, Dublin, Ireland). Samples were immediately refrigerated at 4 °C and centrifuged within 12 h of collection. Serum from each wild pig was aliquoted into 2 ml Corning^®^ cryovials (Corning Incorporated, Lowell, Massachusetts, USA) and refrigerated for up to 4 days prior to shipment on ice packs to a designated National Animal Health Laboratory Network facility (see serological analyses subsection). For population genetic analyses, we collected an additional 0.5 ml whole blood from 301 of the 348 animals by cardiac puncture or orbital draw. The sample was stored immediately in 1 ml mammalian lysis buffer (Qiagen, Valencia, CA, USA) on ice packs prior to refrigeration at 4 °C. Due to logistic constraints, we collected additional whole blood from only a subset of the individuals. From the remaining 47 animals, we collected hair, which was stored in paper envelopes in the field. Both whole blood and hair samples were transported to the University of Florida and stored at − 80 °C until DNA could be extracted. The University of Florida’s Institutional Animal Care and Use Committee approved the protocol for this study.

### Serological Analyses

Serological tests indicated the presence or absence of host antibodies to a pathogen. Because both PrV and *Brucella* spp. induce a lifelong infection and antibody production, a positive antibody test indicated that an animal was either previously exposed and infected but not currently infected, or exposed and infected. Seroprevalence data for PrV and *Brucella* spp. have previously been used to determine disease prevalence and risk of transmission in wildlife populations (Cross et al. 2007; Pannwitz et al. 2012). We included serological data for wild pigs if the sample was unequivocally determined to be either seropositive or seronegative for PrV and/or *Brucella* spp., and the sex and age of the animal were known. Serological tests were performed at the Kentucky Federal Brucellosis Laboratory (KY-FBL). Sera were screened for PrV using the PrV-gB enzyme-linked immunosorbent assay per the manufacturer’s recommendations (ELISA; Idexx Laboratories, Westbrook, Maine, USA). Samples with S/N ratios ≤ 0.6 were determined as PrV-seropositive, while samples with values > 0.7 were considered as PrV-seronegative. Sera were screened for *Brucella* spp. using the fluorescence polarization assay (FPA), as described by Nielsen et al. (1999). Samples with a result of 20 millipolarization units or above were determined as *Brucella* spp.-seropositive, while samples with values < 20 were considered as *Brucella* spp.-seronegative.

### DNA Isolation and Microsatellite Genotyping

We extracted DNA from blood or hair using the Qiagen DNeasy Blood and Tissue Kit (Qiagen, Valencia, CA, USA) or the QIAamp DNA Micro Kit (Qiagen, Valencia, CA, USA), respectively. For both procedures, we followed the manufacturer’s protocol, with slight modifications reported previously (see Hernández et al. 2018). We stored isolated DNA at − 20 °C. Sixty-one microsatellite markers were initially selected for multilocus genotyping and have been previously described (Ellegren et al. 1993; Robic et al. 1994; Alexander et al. 1996; Rohrer et al. 1996; Hernández et al. 2018). Ultimately, 52 markers were multiplexed for PCR (Hernández et al. 2018). We analyzed PCR products by capillary electrophoresis on an ABI 3130xl Genetic Analyzer (Applied Biosystems, Foster City, CA, USA) and scored using GeneMarker version 2.6.2 (SoftGenetics, State College, PA, USA) at the University of Florida. Validation of genotypes, comparison of genotypes from different biosamples, and calculation of genotyping error have been previously reported (Hernández et al. 2018).

### Population Genetic and Dispersal Analyses

To quantify the level of genetic differentiation (i.e., genetic distance) across wild pig sampling locations, we calculated the overall *F*_ST_ across all genotypes and loci, and their statistical significance tested by 999 permutations using the G-statistic Monte Carlo test implemented in the R package hierfstat 0.04-26 (Goudet 2005). We also calculated pairwise *F*_ST_ values between sampling locations (Weir and Cockerham 1984) and their statistical significance determined by 999 permutation using GenAlEx version 6.5 (Peakall and Smouse 2012).

We estimated population-wide dispersal among locations in the Kissimmee River Valley by estimating the mean posterior proportion of individuals that migrated between each pair of locations. We calculated 95% credible intervals (CI) for pairwise migration estimates between sampling locations, considering credible intervals that did not include zero to be statistically significant.

We identified the individuals within those locations that were either first- or second-generation immigrants, measured as individual ancestry. This parameter estimated the probability that an individual originated from a different location (first-generation immigrant) or was an F1 descendant of the immigrant and a local animal (second-generation immigrant). To estimate the probabilities, we ran 100,000,000 Markov chain Monte Carlo iterations of a model that characterized changes in gene frequencies across populations due to migration. We used a 10,000,000-step burn-in period and a sampling interval of 500 steps. We tested multiple delta values for the mixing parameters of migration, allele frequencies and inbreeding values, where delta values were defined as the maximum amount a parameter could be changed between each iteration. Delta values set to 1 resulted in optimal acceptance rates for changes to each mixing parameter (between 20 and 60%). We conducted multiple runs initialized with dispersed starting values and compared the posterior mean parameter estimates for convergence. Migration rates and individual probability of being an immigrant were estimated using BAYESASS version 3.0 (Wilson and Rannala 2003).

### Land Cover Categorization

Land cover data were categorized into six cover types: (1) closed canopy hardwood forest; (2) open canopy pine, prairie, and scrub (hereafter referred to as “open canopy”); (3) freshwater wetland; (4) lake and river; (5) agriculture; and (6) anthropogenic cover (Table 1). We created these broad-scale groups using the Cooperative Land Cover v3.2 Raster layer (10 m^2^ resolution derived from aerial photography, ground-truthing, and local knowledge; FWC and FNAI 2016) pursuant to the classification schemes described by Anderson et al. (1976), Knight et al. (2010) and Kawula (2014). We estimated the available land cover at each sampling site within a uniform spatial buffer with a radius of 5.75 km (area = 103.9 km^2^). This area encompassed the average home range size of wild pigs in the Gulf Coast forest habitat of the southeastern United States (mean = 4.8 km^2^, Garza et al. 2018) and thus represented the heterogeneity of the biophysical environments wild pigs might encounter within its home range. As such, this area represented a conservative proxy of the landscape scale where pathogens may interact with hosts across the studied ecological system (Meentemeyer et al. 2012). For sites where the exact location of wild pigs at time of death was unknown (i.e., where sampling occurred at hunter check stations), we placed the buffer around the geographic center of the managed area per the boundaries provided by the Florida Conservation Lands (FLMA) shapefile (FNAI 2016). For sites with exact geographic data for samples (i.e., where sampling occurred at the location of euthanasia), we overlaid the buffer around the geographic center of the site’s cluster of sampling points. The buffered area was then assessed for the proportion of the six land cover types, and the same measures of proportional land cover were assigned to all individuals within a given site. All spatial data collection was performed in ArcMap 10.4.1 (ESRI 2016).

**Table 1:** Classification Scheme of Land Cover Types Relevant to Wild Pig Biology.

Hardwood forest	Pine, prairie, and scrub (open canopy)	Freshwater wetland	Lake and river	Agriculture	Anthropogenic
Upland hardwood forest	High pine and scrub	Freshwater non-forested wetlands	Lacustrine	Cropland/pasture	Cultural-terrestrial
Mesic hammock	Scrub	Prairies and bogs	Natural lakes and ponds	Orchards/groves	Rural
Slope forest	Sand pine scrub	Marshes	Cultural—lacustrine	Vineyard and nurseries	Extractive
Xeric hammock	Coastal scrub	Isolated freshwater marsh	Riverine	Other agriculture	Bare soil/clear cut
Mixed hardwood-coniferous	Upland pine	Floodplain marsh	Natural rivers and streams	Improved pasture	Low-intensity urban
Maritime hammock	Sandhill	Freshwater forested wetlands	Cultural—riverine	Sugarcane	High-intensity urban
	Pine flatwoods and dry prairie	Cypress/tupelo (including Cypress/tupelo mixed)			Transportation
	Dry flatwoods	Cypress			Communication
	Mesic flatwoods	Isolated freshwater swamp			Utilities
	Scrubby flatwoods	Strand swamp			
	Dry prairie	Floodplain swamp			
	Palmetto prairie	Other coniferous wetlands			
	Shrub and brushland	Wet flatwoods			
	Tree plantations	Other hardwood wetlands			
		Baygall			
		Hydric hammock			
		Non-vegetated wetland			
		Cultural—palustrine			
		Dome swamp			
		Basin swamp			

Fine-scale land cover types drawn from the Cooperative Land Cover v3.2 Raster—State Classes layer (FWC and FNAI 2016).

### Predictors of PrV and *Brucella* spp. Exposure in Wild Pigs

Within an Akaike information criterion (AIC) framework for model comparison (Burnham and Anderson 2002), we used logistic mixed effect regression models to assess the effect of the probability of recent (first or second-generation) individual migration, the proportion of each land cover type, age class (juvenile, sub-adult, or adult), and sex (male or female) on the odds of PrV and *Brucella* spp. exposure (seropositive = 1, seronegative = 0). We also included two-way interactions of agriculture with the other five land cover types to assess the influence of agricultural expansion on the relationship between PrV and *Brucella* spp. exposure and non-agricultural land cover. Prior to their inclusion in the models, predictor variables were tested for collinearity using Pearson correlation coefficients, and we found no terms exceeding the 0.7 threshold (Booth et al. 1994). To account for heterogeneity of PrV and *Brucella* spp. exposure across sampling sites, we included a random site-specific intercept in all models. Exploratory analyses indicated that including the random site effect significantly (*p* < 0.001) improved the overall model fit over the fixed-effects model (which included variables of migration, land cover, age class, sex and two-way interactions between land cover types). However, because the land cover types were spatially varying along with the site-specific random effect, the estimates for the regression coefficients of the fixed effects were confounded with the random intercepts. To alleviate the confounding of land cover with the random effects, we projected the random effects into the null space of the land cover variables so that the site-specific intercepts only accounted for variation not already explained by land cover (Reich et al. 2006). We fit all models and calculated regression coefficients and 95% confidence intervals (CIs) using the R package mgcv v1.8-16 (Wood 2006) and performed a likelihood ratio test as a measure of goodness of fit using the R package lmtest v0.9-36 (Zeileis and Hothorn 2002). Odds ratios for all variables were calculated by exponentiating the logistic regression coefficients, and statistical significance was determined as a 95% CI that did not include one.

## Results

### Population Genetic and Dispersal Analyses

The overall *F*_ST_ = 0.09 was statistically significant across all genotypes and loci (G-statistic = 26,334.4, *p* < 0.05). All pairwise F_ST_ values estimated between sampling locations were significantly different from zero (*p* < 0.05), which indicated genetic differentiation among sampling locations. *F*_ST_ values ranged from 0.020 (between locations 9 and 17) to 0.165 (between locations 1 and 2). Ten of 24 sampling sites showed moderate levels of genetic differentiation (all *F*_ST_ values > 0.05) compared to the rest of sampling sites (see Online Resource 1).

Analysis of dispersal patterns via estimation of migration rates revealed low and statistically insignificant migration among most sampling locations. However, we found significant mean posterior proportion of individuals that migrated between one site (location 17) and 15 other adjacent sampling sites throughout the Kissimmee Valley (ranging from 4 to 14% migrants between sites) (see Online Resource 2). For locations that had significant migration rates between them and location 17, we identified 130 of 156 wild pigs that exhibited a probability > 0.9 to be either first or second-generation immigrant from a source location different than the sampling location.

### Predictors of PrV and *Brucella* spp. Exposure

Total sample sizes of wild pigs after omissions were 297 (25 juveniles, 24 sub-adults, 248 adults) for PrV exposure, and 291 (24 juveniles, 22 sub-adults, 245 adults) for *Brucella* spp. We observed roughly equivalent sex ratios within both sample populations (PrV: 148 males, 149 females; *Brucella* spp.: 146 males, 145 females). PrV-seropositive animals were detected at 21 of 23 sites (91.3%), and *Brucella* spp.-seropositive individuals were found at 14 of 23 sites (60.9%). Within each sampled population, 166 (55.9%; CI 50.0–61.6%) exhibited PrV-specific antibodies in their serum, and 35 (12.0%; CI 8.5–16.3%) exhibited *Brucella* spp.-specific antibodies.

**Figure 2:**
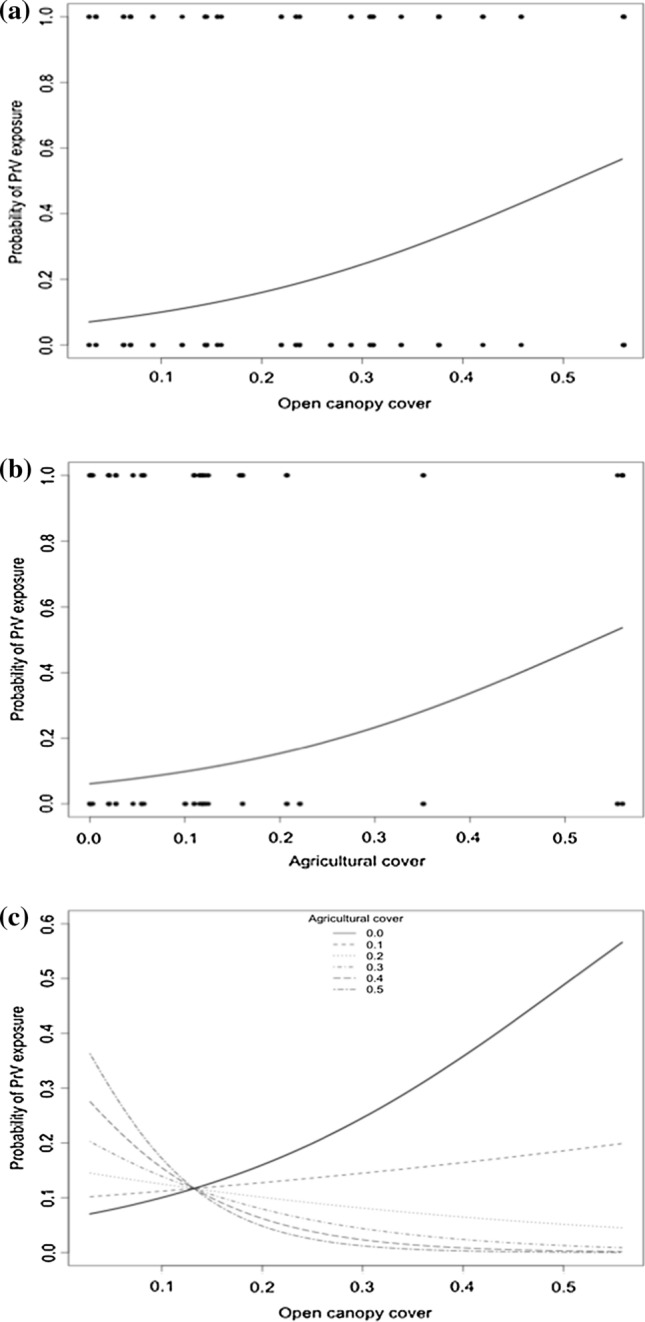
Main effects of (**a**) open canopy cover (pine, prairie, and scrub) and (**b**) agricultural cover (crop and pasture) proportions, and (**c**) two-way interaction between open canopy with agricultural as predictors of the probability of pseudorabies virus (PrV) exposure in wild pigs. In Figure 2c, X-axis and top legend depict the range of open canopy and agricultural cover proportions, respectively, estimated across 23 collection sites of the Kissimmee Valley of Florida.

We summarized the PrV and Brucella spp. seroprevalences, and the probability of recent migration and proportion of each land cover type as predictors of pathogen exposure across sampling locations (see Online Resource 3). The best-ranked AIC model predicting PrV exposure included the probability of recent migration, age class, open canopy, agriculture, and the interaction between agriculture and open canopy (Table 2). Odds of PrV exposure were over three times higher (odds ratio [OR] = 3.25) for recent migrant than for non-migrant wild pigs, and almost four times higher (OR = 3.56) for adults than for juveniles (Table 3). Odds of PrV exposure were also over 100 times higher for wild pigs on lands dominated by open canopy (OR = 214.86) and agriculture (OR = 170.72) than wild pigs in areas without these land cover types. Though both open canopy and agriculture had positive main effects on PrV exposure, the effect of open canopy on PrV exposure became increasingly negative as agricultural cover increased (Fig. 2). Both null and fixed-effects-only models exhibited ΔAIC > 2, and likelihood ratio tests confirmed that the best-ranked AIC model fit the PrV data significantly better than the null model (*χ*^2^ = 89.50, *df* = 18.10, *p* < 0.001) and the fixed-effects-only model (*χ*^2^ = 28.05, *df* = 3.10, *p* < 0.001). None of the remaining variables (sex, hardwood forest, freshwater wetland, lake and river, or anthropogenic land covers) were significantly related to changes in PrV exposure across all the candidate logistic regression models.

**Table 2:** AIC-Ranking of Candidate Logistic Regression Models as Predictors of the Probabilities of Pseudorabies Virus (PrV) and *Brucella* spp. Exposure, Respectively.

Pathogen	Model	*K*^a^	ΔAIC^b^	$R^{2}_{\text{adj}}$
PrV	Migration + Age + OC^c^ + AG^d^ + OC × AG + Site	8	0.00	0.22
	Migration + Age + FW^e^ + OC + AG + OC × AG + Site	9	0.54	0.22
	Migration + Age + Sex + HF^f^ + OC + FW + LR^g^ + AG + AN^h^ + HF × AG + OC × AG + FW × AG + LR × AG + AN × AG	16	21.86	0.14
	Null model	2	53.31	0
*Brucella* spp.	Migration + site	4	0.00	0.11
	Null model	2	13.02	0
	Migration + Age + Sex + HF + OC + FW + LR + AG + AN + HF × AG + OC × AG + FW × AG + LR × AG + AN × AG	16	13.62	0.06

Only models with ΔAIC < 2, and null and fixed-effects-only models are presented

^a^Number of estimable parameters

^b^Difference in AIC between given model and model with minimum AIC

^c^Open canopy (pine, prairie, and scrub)

^d^Agriculture

^e^Freshwater wetland

^f^Hardwood forest

^g^Lake and river

^h^Anthropogenic

**Table 3:** Summary of the Predictors of Pseudorabies Virus (PrV) and *Brucella* spp. Exposure, Respectively.

Pathogen	Parameter	OR	95% CI_OR_
PrV	Intercept^a^	0.07	(0.02, 0.25)
	Migration^a^	3.25	(1.70, 6.23)
	Sub-adult age class	0.84	(0.23, 3.00)
	Adult age class^a^	3.56	(1.35, 9.30)
	Open canopy (OC)^a^	214.86	(22.20, 2079.74)
	Agriculture (AG)^a^	170.72	(6.69, 4359.01)
	AG × OC^a^	1.26e^−17^	(1.44e^−28^, 1.11e^−6^)
*Brucella* spp.	Intercept^a^	0.08	(0.04, 0.14)
	Migration	2.23	(0.99, 5.00)

Odds ratio (OR) and 95% confidence interval (95% CI_OR_) for each predictor are presented. Parameter values of the best-ranked AIC models are presented.

^a^Variables with significant confidence intervals (95% CI_OR_)

The best-ranked AIC model predicting *Brucella* spp. exposure only included the probability that an individual was a first- or second-generation immigrant (Table 2). The odds of *Brucella* spp. exposure (odds ratio [OR] = 2.23) did not increase significantly for wild pigs that were recent immigrants (Table 3), but the 95% CI_OR_ marginally included one. None of the remaining variables (any land cover class, age class, or sex) was a significant predictor of *Brucella* spp. exposure.

## Discussion

Our findings suggest that wild pig dispersal and landscape composition influence pathogen exposure for PrV among wild pigs throughout the Kissimmee Valley of Florida. First- and second-generation immigrants contributed to increased PrV seroprevalence in wild pig populations, likely due to increased contact among infectious and susceptible individuals. Similarly, agriculture-dominated areas, which attract high densities of wild pigs due to the availability of abundant artificial food resources, had higher PrV seroprevalence than other land cover types. Contrary to expectations, the odds of PrV exposure were also higher at sites dominated by the resource-limited open canopy habitat. The mechanism driving these two apparently contradictory relationships may be the patchiness of resources within both land cover types. Indeed, the effect of open canopy on PrV exposure reversed when agricultural cover was available, suggesting that local distribution of resources may play a role in pathogen transmission. These results underscore the necessity for large-scale sampling both within and among populations to elucidate landscape-level disease dynamics and interactions among driving factors.

The role of movement in PrV exposure observed here corroborates previous observations that dispersal (both natural and anthropogenic) increases contact rates between pathogen-exposed and susceptible individuals, which contributes to disease spread at the population level (e.g., Zanardi et al. 2003; Hampton et al. 2006; Keuling et al. 2008; Pearson et al. 2014; Franckowiak and Poché 2018). In Florida, wild pig dispersal has been strongly influenced by successive events of human-assisted movement that have contributed to the geographical expansion of wild pigs throughout the Kissimmee Valley and adjacent regions (Hernández et al. 2018). Escapes from holding facilities, legal and illegal transport and release, and hunting pressures have contributed to the movement of wild pigs into areas that have less human disturbance or are unoccupied by wild pig social groups (Zanardi et al. 2003; Keuling et al. 2008). In the present study, much of the pattern of movement was driven by movement of animals into and out of location 17, which was within close proximity to a private hunting club known to transport animals into and out of the property. This anthropogenically induced movement likely resulted in the high levels of admixture and production of *F*1/*F*2 individuals from the mating between animals from location 17 and other source populations (see Hernández et al. 2018 for details), potentially affecting the opportunity for contact between naïve and infectious individuals, as suggested by previous studies (e.g., Zanardi et al. 2003; Cowled and Garner 2008). Because the movement of individual wild pigs transcended property boundaries and was facilitated by human-assisted movement, land managers wishing to control the spread of PrV among wild pigs, and from wild pigs to livestock and native wildlife, may benefit from cooperative efforts among public agencies and private landowners as well as from enforcement of animal movement laws in the state.

In addition to an animal’s dispersal history, the increase in PrV exposure was also associated with an increase in the proportion of both agricultural and open canopy cover within an animal’s home range. This finding suggests that small-scale spatial and temporal distribution of resources may be more important to the spread of directly transmitted diseases than overall resource abundance. Agricultural lands offer reliable access to diverse crop types (Genov 1981; Herrero et al. 2006), artificial water sources (Carrasco-Garcia et al. 2015; Payne et al. 2015) and supplemental feeding areas (Cross et al. 2007; Campbell et al. 2013). The availability of these resources not only drives overall wild pig densities up to four times higher in cropland and pasture than in surrounding habitat types (Caley 1993; Kay et al. 2017), but also attracts higher animal concentrations around the individual sources of food and water, creating local conditions for heightened exposure to directly transmitted pathogens. In contrast, water, thermal refugia, and highly preferred food resources are limited in open canopy (i.e., pine, prairie, and scrub) habitat, relative to hardwood forest and freshwater wetland habitats (Mayer and Brisbin Jr 2009; Saito et al. 2012; Keiter and Beasley 2017). However, the temporal patchiness of resources, either as food (Kurz and Marchinton 1972; Hughes 1985) or refugia from hunting (Gaston et al. 2008; Franckowiak and Poché 2018), within open canopy habitat may mimic the supplemental resources provided by agricultural areas in elevating local concentrations of wild pigs around discrete resources. This apparent role of small-scale distribution of resources in modulating disease transmission is bolstered by our finding that increasing agricultural cover reversed the effect of open canopy on PrV exposure. The local presence of artificial food and water sources may have allowed wild pigs to commute to agricultural fields from less attractive and sparse nutritional conditions in open canopy habitat, as demonstrated by previous studies (Gerard et al. 1991; Schley and Roper 2003; Herrero et al. 2006; Keuling et al. 2009). Consequently, contact and therefore pathogen exposure may be reduced among animals within the adjacent open canopy. Combined with the effects of wild pig dispersal, these results suggest that the processes driving pathogen exposure operate at a variety of spatial and temporal scales, reiterating the need for collaborative management efforts in controlling the spread of this non-native pathogen to species of conservation concern.

We detected no influence of land cover composition on *Brucella* spp.; while we found that wild pig dispersal weakly related to *Brucella* spp. exposure, we may be tempted to interpret recent migration as contributing to the persistence of this bacterial agent among wild pig populations. However, the relatively low number of wild pigs exposed to *Brucella* spp. (12%; 35/291) likely impeded our power to detect any significant predictor variables from the models. There are several known limitations of existing serological diagnostic tests for *Brucella* spp. (e.g., limited sensitivity and specificity and cross-reactivity with other pathogens) that tend to underestimate the proportion of wild pigs exposed to the bacteria (Pedersen et al. 2014, 2017). Larger sample sizes using improved serological tests would facilitate our understanding of the roles of animal movement and land cover for this pathogen.

While we suggest that host dispersal and resource-driven contacts predict the likelihood of PrV exposure among wild pigs, our study has methodological caveats that warrant a more cautious interpretation of results. First, considering that population density modulates the infection dynamics of several pathogens due to its impact on contact rates (Penrith et al. 2011; Pearson et al. 2016), variability in host population density may act as a potential confounding variable that influences PrV transmission between infected and susceptible individuals (e.g., Cowled et al. 2012). Unfortunately, lack of wild pig density data prevented us to include an independent measure of host density as potential predictor of pathogen exposure in our statistical models (i.e., there are no systematic records of hunting bag numbers across hunter check stations or public/private properties in the state of Florida). Second, the extreme odds of PrV exposure related to landscape composition were likely caused by the imbalanced number of animals opportunistically sampled across sites (range: 3–45 wild pigs per site after omissions), which may limit our inferences about the contribution of land cover on the risk of PrV spread among wild pigs.

Because of the risk of pathogen spill-over to other species, the findings presented here have direct implications to carnivore conservation. Movement of wild pigs infected with directly transmitted or water-borne pathogens has the potential to increase the risk of infection for other sympatric species (Hampton et al. 2006; Franckowiak and Poché 2018), and PrV is highly lethal to mammalian carnivores (Stallknecht and Howerth 2008). The results of this study suggest that patterns of wild pig dispersal and land cover composition may be used to predict habitats that present a high risk for cross-species transmission of PrV to endangered carnivores such as the Florida panther. Panthers are highly susceptible to this pig-borne pathogen (Glass et al. 1994), yet pigs represent the largest component of the Florida panther’s diet (Maehr et al. 1990). More globally, our approach could be extrapolated to understand which habitats within sympatric distributions of wild pigs and susceptible carnivores have a high risk of transmission and used to guide habitat management for endangered carnivores, such as the Iberian lynx (*Lynx lynx,Masot et al.* 2016) and European wolf (*Canis lupus,* Verpoest et al. 2014). Future studies may also test alternative hypotheses, such as the role of stress and its immunosuppressive effects as potential stimuli for increased disease transmission among wild pigs (Allwin et al.^2015^, ^2016^) and from wild pigs to endangered carnivore species. Finally, future landscape epidemiology studies should embrace multiscale data collection and analytical methods to better understand how host and landscape ecology influence the spread and persistence of pathogens across heterogeneous landscapes (Meentemeyer et al. 2012).

## Electronic supplementary material

Below is the link to the electronic supplementary material.

Supplementary material 1 (DOC 89 kb)

Supplementary material 2 (DOC 36 kb)

Supplementary material 3 (PDF 87 kb)
