# Supplementary material for: Dispersal and Land Cover Contribute to Pseudorabies Virus Exposure in Invasive Wild Pigs
Source: Ecohealth. 2021 Jan 14;17(4):498–511. doi: 10.1007/s10393-020-01508-6 (PMC8192353; doi:10.1007/s10393-020-01508-6)
Supplement: Supplementary file 1 — Supplementary material 1 (DOC 89 kb) [file 10393_2020_1508_MOESM1_ESM.doc]

**Article title:**

Dispersal and land cover as contributing factors of pathogen exposure in invasive wild pigs (*Sus scrofa*)

**Journal name:**

Ecohealth

**Author names:**

Felipe A. Hernández, Amanda N. Carr, Michael P. Milleson, Hunter R. Merrill, Michael L. Avery, Brandon M. Parker, Cortney L. Pylant, James D. Austin, Samantha M. Wisely

**Affiliation and e-mail address of the corresponding author:**

School of Natural Resources and Environment, University of Florida, 103 Black Hall, PO Box 116455, Gainesville, Florida 32611, USA

Department of Wildlife Ecology and Conservation, University of Florida, 110 Newins-Ziegler Hall, PO Box 110430, Gainesville, Florida 32611, USA

wisely@ufl.edu

**Online Resource 1**

Pairwise estimates of F_ST_ values among sampling locations of wild pigs (below diagonal) and their corresponding *p*-values (all significant *p*<0.01; above diagonal)

| **Location** | **1** | **2** | **3** | **4** | **5** | **6** | **7** | **8** | **9** | **10** | **11** | **12** | **13** | **14** | **15** | **16** |
| --- | --- | --- | --- | --- | --- | --- | --- | --- | --- | --- | --- | --- | --- | --- | --- | --- |
| 1 |  | 0.001 | 0.003 | 0.001 | 0.003 | 0.001 | 0.001 | 0.001 | 0.001 | 0.003 | 0.001 | 0.001 | 0.001 | 0.001 | 0.001 | 0.002 |
| 2 | 0.165 |  | 0.001 | 0.001 | 0.002 | 0.001 | 0.001 | 0.001 | 0.001 | 0.001 | 0.001 | 0.001 | 0.001 | 0.001 | 0.001 | 0.001 |
| 3 | 0.138 | 0.140 |  | 0.001 | 0.003 | 0.001 | 0.001 | 0.001 | 0.001 | 0.002 | 0.001 | 0.001 | 0.001 | 0.001 | 0.001 | 0.003 |
| 4 | 0.111 | 0.135 | 0.095 |  | 0.001 | 0.001 | 0.001 | 0.001 | 0.001 | 0.001 | 0.001 | 0.001 | 0.001 | 0.001 | 0.001 | 0.001 |
| 5 | 0.123 | 0.132 | 0.098 | 0.059 |  | 0.001 | 0.001 | 0.001 | 0.001 | 0.002 | 0.001 | 0.001 | 0.001 | 0.002 | 0.001 | 0.005 |
| 6 | 0.114 | 0.118 | 0.091 | 0.057 | 0.069 |  | 0.007 | 0.001 | 0.001 | 0.001 | 0.001 | 0.001 | 0.001 | 0.001 | 0.001 | 0.001 |
| 7 | 0.131 | 0.113 | 0.083 | 0.069 | 0.072 | 0.055 |  | 0.001 | 0.001 | 0.001 | 0.001 | 0.001 | 0.001 | 0.001 | 0.001 | 0.001 |
| 8 | 0.126 | 0.136 | 0.103 | 0.065 | 0.069 | 0.076 | 0.076 |  | 0.001 | 0.001 | 0.001 | 0.001 | 0.001 | 0.001 | 0.001 | 0.001 |
| 9 | 0.095 | 0.103 | 0.067 | 0.040 | 0.048 | 0.043 | 0.046 | 0.046 |  | 0.001 | 0.001 | 0.001 | 0.001 | 0.001 | 0.001 | 0.001 |
| 10 | 0.143 | 0.150 | 0.113 | 0.093 | 0.093 | 0.101 | 0.089 | 0.090 | 0.067 |  | 0.001 | 0.002 | 0.001 | 0.001 | 0.001 | 0.002 |
| 11 | 0.099 | 0.105 | 0.071 | 0.055 | 0.057 | 0.047 | 0.052 | 0.050 | 0.029 | 0.070 |  | 0.001 | 0.001 | 0.001 | 0.001 | 0.001 |
| 12 | 0.131 | 0.146 | 0.091 | 0.081 | 0.078 | 0.078 | 0.082 | 0.087 | 0.061 | 0.105 | 0.066 |  | 0.001 | 0.001 | 0.001 | 0.002 |
| 13 | 0.133 | 0.148 | 0.119 | 0.080 | 0.087 | 0.074 | 0.084 | 0.083 | 0.057 | 0.108 | 0.058 | 0.098 |  | 0.001 | 0.001 | 0.001 |
| 14 | 0.126 | 0.138 | 0.088 | 0.067 | 0.068 | 0.064 | 0.072 | 0.073 | 0.047 | 0.091 | 0.056 | 0.085 | 0.092 |  | 0.001 | 0.002 |
| 15 | 0.145 | 0.132 | 0.119 | 0.093 | 0.094 | 0.078 | 0.089 | 0.086 | 0.065 | 0.097 | 0.067 | 0.099 | 0.103 | 0.091 |  | 0.001 |
| 16 | 0.141 | 0.150 | 0.141 | 0.088 | 0.109 | 0.093 | 0.101 | 0.099 | 0.084 | 0.123 | 0.082 | 0.124 | 0.109 | 0.103 | 0.123 |  |
| 17 | 0.086 | 0.089 | 0.061 | 0.045 | 0.046 | 0.039 | 0.044 | 0.043 | 0.020 | 0.062 | 0.022 | 0.053 | 0.056 | 0.046 | 0.055 | 0.076 |
| 18 | 0.112 | 0.139 | 0.096 | 0.068 | 0.070 | 0.068 | 0.075 | 0.075 | 0.042 | 0.094 | 0.059 | 0.087 | 0.089 | 0.071 | 0.091 | 0.105 |
| 19 | 0.111 | 0.114 | 0.081 | 0.066 | 0.066 | 0.058 | 0.067 | 0.063 | 0.039 | 0.091 | 0.044 | 0.071 | 0.088 | 0.065 | 0.076 | 0.101 |
| 20 | 0.106 | 0.102 | 0.077 | 0.057 | 0.061 | 0.057 | 0.059 | 0.053 | 0.034 | 0.075 | 0.040 | 0.064 | 0.075 | 0.052 | 0.070 | 0.085 |
| 21 | 0.150 | 0.156 | 0.114 | 0.088 | 0.093 | 0.094 | 0.101 | 0.103 | 0.067 | 0.120 | 0.078 | 0.120 | 0.117 | 0.088 | 0.118 | 0.133 |
| 22 | 0.104 | 0.110 | 0.078 | 0.058 | 0.067 | 0.063 | 0.067 | 0.058 | 0.035 | 0.078 | 0.043 | 0.070 | 0.074 | 0.063 | 0.083 | 0.085 |
| 23 | 0.123 | 0.140 | 0.104 | 0.069 | 0.077 | 0.067 | 0.080 | 0.076 | 0.048 | 0.094 | 0.051 | 0.092 | 0.084 | 0.084 | 0.095 | 0.104 |
| 24 | 0.134 | 0.158 | 0.111 | 0.093 | 0.111 | 0.096 | 0.111 | 0.105 | 0.074 | 0.124 | 0.075 | 0.116 | 0.114 | 0.104 | 0.122 | 0.142 |

| **Location** | **17** | **18** | **19** | **20** | **21** | **22** | **23** | **24** |
| --- | --- | --- | --- | --- | --- | --- | --- | --- |
| 1 | 0.001 | 0.006 | 0.001 | 0.001 | 0.005 | 0.001 | 0.001 | 0.001 |
| 2 | 0.001 | 0.001 | 0.001 | 0.001 | 0.001 | 0.001 | 0.001 | 0.001 |
| 3 | 0.001 | 0.006 | 0.001 | 0.001 | 0.007 | 0.001 | 0.001 | 0.001 |
| 4 | 0.001 | 0.001 | 0.001 | 0.001 | 0.001 | 0.001 | 0.001 | 0.001 |
| 5 | 0.001 | 0.002 | 0.001 | 0.001 | 0.012 | 0.001 | 0.001 | 0.001 |
| 6 | 0.001 | 0.016 | 0.001 | 0.001 | 0.002 | 0.001 | 0.001 | 0.001 |
| 7 | 0.001 | 0.002 | 0.001 | 0.001 | 0.005 | 0.001 | 0.001 | 0.001 |
| 8 | 0.001 | 0.001 | 0.001 | 0.001 | 0.001 | 0.001 | 0.001 | 0.001 |
| 9 | 0.001 | 0.003 | 0.001 | 0.001 | 0.001 | 0.001 | 0.001 | 0.001 |
| 10 | 0.001 | 0.003 | 0.001 | 0.001 | 0.002 | 0.001 | 0.001 | 0.001 |
| 11 | 0.001 | 0.001 | 0.001 | 0.001 | 0.001 | 0.001 | 0.001 | 0.001 |
| 12 | 0.001 | 0.001 | 0.001 | 0.001 | 0.001 | 0.001 | 0.001 | 0.001 |
| 13 | 0.001 | 0.001 | 0.001 | 0.001 | 0.001 | 0.001 | 0.001 | 0.001 |
| 14 | 0.001 | 0.001 | 0.001 | 0.001 | 0.002 | 0.001 | 0.001 | 0.001 |
| 15 | 0.001 | 0.001 | 0.001 | 0.001 | 0.001 | 0.001 | 0.001 | 0.001 |
| 16 | 0.001 | 0.006 | 0.001 | 0.001 | 0.005 | 0.001 | 0.001 | 0.001 |
| 17 |  | 0.003 | 0.001 | 0.001 | 0.001 | 0.001 | 0.001 | 0.001 |
| 18 | 0.048 |  | 0.001 | 0.001 | 0.031 | 0.003 | 0.001 | 0.001 |
| 19 | 0.028 | 0.070 |  | 0.001 | 0.001 | 0.001 | 0.001 | 0.001 |
| 20 | 0.028 | 0.061 | 0.049 |  | 0.001 | 0.001 | 0.001 | 0.001 |
| 21 | 0.071 | 0.094 | 0.099 | 0.080 |  | 0.001 | 0.001 | 0.001 |
| 22 | 0.032 | 0.064 | 0.051 | 0.041 | 0.087 |  | 0.001 | 0.001 |
| 23 | 0.050 | 0.071 | 0.078 | 0.054 | 0.095 | 0.059 |  | 0.001 |
| 24 | 0.067 | 0.100 | 0.080 | 0.084 | 0.134 | 0.081 | 0.106 |  |
