# Supplementary material for: Dispersal and Land Cover Contribute to Pseudorabies Virus Exposure in Invasive Wild Pigs
Source: Ecohealth. 2021 Jan 14;17(4):498–511. doi: 10.1007/s10393-020-01508-6 (PMC8192353; doi:10.1007/s10393-020-01508-6)
Supplement: Supplementary file 2 — Supplementary material 2 (DOC 36 kb) [file 10393_2020_1508_MOESM2_ESM.doc]

**Article title:**

Dispersal and land cover as contributing factors of pathogen exposure in invasive wild pigs (*Sus scrofa*)

**Journal name:**

Ecohealth

**Author names:**

Felipe A. Hernández, Amanda N. Carr, Michael P. Milleson, Hunter R. Merrill, Michael L. Avery, Brandon M. Parker, Cortney L. Pylant, James D. Austin, Samantha M. Wisely

**Affiliation and e-mail address of the corresponding author:**

School of Natural Resources and Environment, University of Florida, 103 Black Hall, PO Box 116455, Gainesville, Florida 32611, USA

Department of Wildlife Ecology and Conservation, University of Florida, 110 Newins-Ziegler Hall, PO Box 110430, Gainesville, Florida 32611, USA

wisely@ufl.edu

**Online Resource 2**

Significant migration rates (% of individuals that migrated between each pair of locations)

| **Locations between which migration occurred** | **Migration rate (mean±s.d.)^a^** |
| --- | --- |
| Location 17 - Location 3 | 6.73±2.2 |
| Location 17 - Location 5 | 6.54±2.2 |
| Location 17 - Location 6 | 4.48±2.18 |
| Location 17 - Location 7 | 5.74±2.4 |
| Location 17 - Location 8 | 8.26±2.66 |
| Location 17 - Location 10 | 7.43±2.31 |
| Location 17 - Location 11 | 14.39±2.4 |
| Location 17 - Location 12 | 8.42±2.45 |
| Location 17 - Location 14 | 9.02±2.85 |
| Location 17 - Location 16 | 6.65±2.21 |
| Location 17 - Location 18 | 4.7±2.08 |
| Location 17 - Location 19 | 7.76±3.2 |
| Location 17 - Location 20 | 13.46±2.33 |
| Location 17 - Location 21 | 5.43±2.5 |
| Location 17 - Location 22 | 11.66±2.58 |

^a^ Migration rates are reported as ±95% credible interval
