# Supplementary material for: Dispersal and Land Cover Contribute to Pseudorabies Virus Exposure in Invasive Wild Pigs
Source: Ecohealth. 2021 Jan 14;17(4):498–511. doi: 10.1007/s10393-020-01508-6 (PMC8192353; doi:10.1007/s10393-020-01508-6)
Supplement: Supplementary file 3 — Supplementary material 3 (PDF 87 kb) [file 10393_2020_1508_MOESM3_ESM.pdf]

Article title:

Dispersal and land cover contribute to pathogen exposure in invasive wild pigs

Journal name:

Ecohealth

Author names:

Felipe A. Hernández, Amanda N. Carr, Michael P. Milleson, Hunter R. Merrill, Michael L. Avery, Brandon M. Parker, Cortney L. Pylant, James D. Austin, Samantha M. Wisely

Affiliation and e-mail address of the corresponding author:

School of Natural Resources and Environment, University of Florida, 103 Black Hall, PO Box 116455, Gainesville, Florida 32611, USA

Department of Wildlife Ecology and Conservation, University of Florida, 110 Newins-Ziegler Hall, PO Box 110430, Gainesville, Florida 32611, USA

wisely@ufl.edu

Online Resource 3

Percentage (95% confidence interval) of wild pigs that exhibited PrV- and *Brucella* spp.-specific antibodies, mean (±SE) probability of recent (first or second-generation) individual migration and proportion of land cover types across 23 sampling locations

Site	PrV	*Brucella* spp.	Individual ancestry	Hardwood forest	Pine, prairie, and scrub (open canopy)	Freshwater wetland	Lake and river	Agriculture	Anthropogenic
1	72.7 (57.2 - 85.0)	4.7 (0.6 - 15.8)	0.19 ± 0.05	0.002	0.560	0.384	0.001	0.020	0.033
2	87.5 (61.7 - 98.4)	17.6 (3.8 - 43.4)	1	0.032	0.035	0.224	0.010	0.560	0.139
3	7.7 (0.2 - 36.0)	7.7 (0.2 - 36.0)	0.15 ± 0.10	0.001	0.145	0.050	0.046	0.000	0.184
4	66.7 (9.4 - 99.2)	0	1	0.032	0.160	0.279	0.017	0.125	0.378
5	55.6 (21.2 - 86.3)	0	0.98 ± 0.01	0.029	0.219	0.284	0.051	0.161	0.255
6	38.5 (13.9 - 68.4)	15.4 (1.9 - 45.4)	0.29 ± 0.11	0.152	0.063	0.163	0.021	0.118	0.483
7	60.0 (14.7 - 94.7)	40.0 (5.3 - 85.3)	1	0.258	0.028	0.540	0.033	0.045	0.096
8	60.0 (44.3 - 74.3)	4.5 (0.6 - 15.5)	0.09 ± 0.03	0.002	0.376	0.574	0.002	0.001	0.045
9	100	60 (14.7 - 94.7)	1	0.186	0.234	0.149	0.010	0.158	0.263
10	23.1 (5.0 - 53.8)	0	0.11 ± 0.08	0.005	0.238	0.364	0.019	0.055	0.314
11	40.0 (12.2 - 73.8)	0	1	0.004	0.289	0.688	0.004	0.000	0.015
12	80.0 (28.4 - 99.5)	0	1	0.008	0.458	0.343	0.000	0.116	0.075
13	70.0 (45.7 - 88.1)	35 (15.4 - 59.2)	0.15 ± 0.07	0.123	0.069	0.382	0.103	0.207	0.116
14	0	0	0	0.107	0.269	0.223	0.032	0.221	0.148
15	64.7 (38.3 - 85.8)	6.3 (0.2 - 30.2)	0.04 ± 0.04	0.067	0.144	0.459	0.044	0.027	0.258
16	41.7 (15.2 - 72.3)	33.3 (9.9 - 65.1)	0.42 ± 0.13	0.115	0.311	0.239	0.004	0.001	0.047
17	20.0 (0.5 - 71.6)	0	0.76 ± 0.05	0.019	0.121	0.246	0.001	0.555	0.058
18	0	0	0.04 ± 0.04	0.185	0.234	0.193	0.086	0.101	0.201
19	83.3 (35.9 - 99.6)	33.3 (4.3 - 77.7)	1	0.018	0.420	0.326	0.014	0.057	0.164
21	40.0 (12.2 - 73.8)	0	1	0.046	0.092	0.367	0.030	0.351	0.114
22	66.7 (22.3 - 95.7)	33.3 (4.3 - 77.7)	1	0.014	0.156	0.775	0.025	0.003	0.028
23	78.9 (54.4 - 93.9)	11.1 (1.4 - 34.7)	1	0.045	0.308	0.307	0.007	0.110	0.222
24	40.0 (5.3 - 85.3)	40.0 (5.3 - 85.3)	1	0.014	0.339	0.169	0.037	0.121	0.216

Values denote predictors of the probability of pseudorabies virus (PrV) exposure after omissions
